# Effects of Paliperidone on Serum D-dimer Levels: Clinical and Experimental Findings

**DOI:** 10.62641/aep.v53i5.1958

**Published:** 2025-10-05

**Authors:** Tingting Mou, Jianbo Lai, Lingzhuo Kong

**Affiliations:** ^1^Department of Psychiatry, The First Affiliated Hospital, Zhejiang University School of Medicine, 310003 Hangzhou, Zhejiang, China; ^2^Zhejiang Key Laboratory of Precision Psychiatry, 310003 Hangzhou, Zhejiang, China; ^3^Brain Research Institute of Zhejiang University, 310012 Hangzhou, Zhejiang, China; ^4^Zhejiang Engineering Center for Mathematical Mental Health, 310003 Hangzhou, Zhejiang, China; ^5^MOE Frontier Science Center for Brain Science and Brain-machine Integration, Zhejiang University School of Medicine, 310058 Hangzhou, Zhejiang, China

**Keywords:** paliperidone, schizophrenia, blood coagulation

## Abstract

**Background:**

Dysregulation of coagulation function associated with antipsychotic treatment remains poorly understood. This study investigates the potential impact of paliperidone on serum D-dimer levels during the early stages of treatment.

**Methods:**

Nine patients diagnosed with first-episode schizophrenic spectrum disorder were assessed for serum D-dimer levels before and after a 2-week paliperidone regimen. Additionally, eight adult C57 mice in the experimental group (EG) received 3 mg/kg of paliperidone daily for 10 consecutive days, while eight mice in the control group (CG) were untreated. Venous blood was collected and analyzed for D-dimer at baseline, and on the 5th and 10th days in the EG, as well as on the 10th day for the CG.

**Results:**

No significant differences were observed in serum D-dimer levels before and after paliperidone treatment in the patient cohort. In animal experiments, compared to the CG on the 10th day, serum D-dimer levels in the EG on the 10th day showed no significant difference (*p >* 0.05), while the level in the EG on the 5th day was significantly lower (*p <* 0.05). Compared to its baseline, serum D-dimer levels within the EG on the 5th day was significantly decreased (*p <* 0.05).

**Conclusion:**

Short-term paliperidone treatment had minimal effects on serum D-dimer levels in both human participants and mice, though transient changes were noted early in treatment. Nonetheless, the potential for drug-induced coagulation disruption should be considered in clinical practice.

## Introduction

Schizophrenic spectrum disorder is a severe and debilitating psychiatric condition, with schizophrenia being the most recognized subtype, affecting approximately 10% of the permanently disabled population [1]. The pathological mechanisms of schizophrenic spectrum disorders are primarily attributed to deficits in sub-cortical dopaminergic neural circuits within the frontal, temporal, and mesostriatal brain regions [2]. This understanding underpins the widespread use of atypical (second-generation) antipsychotics, which function as antagonists at dopamine D2 receptors [3], as the primary therapeutic approach.

Paliperidone (9-hydroxyrisperidone), a major active metabolite of risperidone, is an atypical antipsychotic effective in managing psychotic symptoms across all age groups of patients with schizophrenia. The recommended maintenance dosage ranges from 3 mg to 12 mg per day.

Adverse effects of paliperidone have been well-documented [4]. With increasing clinical experience, attention has turned to the thrombotic events associated with paliperidone. A case study described two patients with schizophrenia experiencing pulmonary thromboembolism linked to long-term moderate doses of paliperidone: a 40-year-old male on 6–8 mg daily for over three years and a 22-year-old male on 9 mg daily for six months [5]. Additionally, acute pulmonary embolism was reported in a 22-year-old patient treated with paliperidone palmitate (100 mg every four weeks) for six months [6]. More recently, a pharmacovigilance study based on the Food and Drug Administration (FDA) Adverse Event Reporting System highlighted a potential association between paliperidone use and venous thromboembolism [7], a finding later reinforced by further analysis [8], raising significant concerns for clinical practice.

In patients with acute psychosis, a hypercoagulable state characterized by elevated levels of D-dimer, P-selectin, and platelet glycoprotein IIb/IIIa receptor expression has been reported [9, 10]. Acute and chronic antipsychotic treatments may have differential effects on coagulation or fibrinolysis in patients with schizophrenia [10]. Notably, a case report described a sharp increase in serum D-dimer levels in a 17-year-old male with schizophrenia during the first week of paliperidone treatment at a dose of 6 mg daily [11]. D-dimer, a fibrin degradation product (FDP), is a sensitive biomarker for thrombotic activity, reflecting ongoing fibrinolysis often secondary to clot formation. Thus, it is clinically useful in monitoring thrombosis, disease progression, and risk stratification [12, 13].

Given the heightened risk of drug-induced hypercoagulability and the need for early intervention to prevent thromboembolic complications, investigating changes in serum D-dimer levels in patients treated with paliperidone is crucial. Moreover, gaining deeper insights into the underlying mechanisms is of great importance. However, limited studies have addressed this issue. This study aims to explore whether paliperidone treatment influences serum D-dimer levels in the early phase and to further investigate the underlying mechanisms.

## Materials and Methods

### Retrospective Clinical Research

#### Participants and Criteria

Medical data from inpatients in the Department of Psychiatry at the First Affiliated Hospital, Zhejiang University School of Medicine, between January 1, 2021, and September 30, 2024, were screened and extracted from the hospital’s electronic medical system. Sampling occurred on October 1, 2024.

#### Inclusion Criteria

(1) Age between 12 and 65 years, with no restrictions on gender or other demographic variables;

(2) Diagnosis of first-episode schizophrenic spectrum disorder, as per the International Statistical Classification of Diseases and Related Health Problems, 10th Revision (ICD-10), with no limit on the total duration of the disease;

(3) No prior exposure to standardized paliperidone or other antipsychotic treatments before admission, and the primary pharmacological intervention post-admission was oral paliperidone tablets, with a daily dosage between 3 mg and 12 mg;

(4) Use of benzodiazepines for sleep aid and bromocriptine for hyperprolactinemia was permitted;

(5) No personal history of thrombosis or other potential thrombosis risk factors;

(6) Accessible medical history;

(7) Baseline fibrinogen equivalent unit (FEU) levels before the initiation of paliperidone intervention must be within the reference range (≤700 µg/L) at our hospital.

#### Exclusion Criteria

(1) Women who are pregnant or breastfeeding;

(2) Presence of other severe co-morbid psychiatric disorders, including bipolar disorder or major depressive disorder;

(3) Personal history or current presence of diseases that directly affect coagulation (e.g., cardiovascular diseases, blood disorders);

(4) Presence of complicating physical conditions (and corresponding medications) that could indirectly affect coagulation function (e.g., infection, malignancy, autoimmune diseases);

(5) Current use of anticoagulants or antiplatelet agents.

#### Intervention and Laboratory Assay

All included patients received standardized paliperidone treatment. The initial daily dosage started at 3 mg and was gradually adjusted to an appropriate maintenance dosage, with a maximum of 12 mg per day. Venous blood samples were collected for serum D-dimer testing. Baseline D-dimer levels were measured on the last day before the first paliperidone dose, and the observation period concluded 14 days after the daily dosage stabilized.

D-dimer levels were determined using an automated, particle-enhanced immunoturbidimetric method with the INNOVANCE D-Dimer assay (No. 02240, Siemens Healthineers Diagnostics, Marburg, Germany), following the manufacturer’s protocol. The assay’s reportable range for D-dimer was 170–4400 µg/L FEU [14].

### Animal Experiments

#### Animals and Chemicals

The animal experiments were conducted on 16 adult male C57 wild-type mice (8–12 weeks old, 25.73 ± 0.85 g), obtained from the Zhejiang Medical Science Experimental Animal Service Center. Mice were housed in a room with controlled temperature (25 ± 5 °C) and humidity (55 ± 5%) under a 12-hour light/dark cycle, with ad libitum access to a commercial standard mouse diet and water. Mice were randomly assigned to the experimental group (EG, *N* = 8) or control group (CG, *N* = 8).

Paliperidone (98% purity) was obtained from Shanghai Macklin Biochemical Technology Co., Ltd. (No. P816926, Shanghai, China), and mouse blood D-dimer test kits were purchased from Wuhan Beinley Biological Co., Ltd. (No. MU30725, Wuhan, China).

#### Intervention and Laboratory Assay

Following a one-week adaptation period, the CG (*N* = 8) received drinking water as a vehicle (0.2 mL), while the EG (*N* = 8) received 3 mg/kg paliperidone orally *via* gavage once daily for 10 consecutive days. The CG did not receive any additional treatment. Blood samples were collected from both groups on the baseline, 5th, and 10th days of the study. Mice in the EG were anesthetized with inhaled isoflurane (1–2% concentration), and blood was drawn from the posterior orbital venous plexus. Mice in the CG were similarly anesthetized and sampled on the 10th day only. After blood collection on the 10th day, all mice were euthanized by cervical dislocation. Blood samples were left to coagulate at room temperature for 30 minutes, followed by centrifugation at 3000 g for 10 minutes. The serum was separated and stored at –80 °C. Serum D-dimer levels in mice were assessed using the sandwich enzyme-linked immunosorbent assay (S-ELISA) (Wuhan Beinley Biotechnology Co., Ltd., No. MU30725, Wuhan, China).

### Statistical Analyses

Data analysis and graph generation were performed using STATA SE Version 17.0 (StataCorp LLC, College Station, TX, USA). Normality of continuous variables was tested using the Shapiro-Wilk test. For normally distributed data, repeated measures analysis of variance (ANOVA) was conducted for multiple comparisons, with post-hoc tests using Bonferroni correction. For non-normally distributed data, the Kruskal-Wallis test was used, with Dunn’s test for post-hoc correction. Independent sample *t*-tests were employed for inter-group comparisons. Data are presented as mean ± standard deviation (SD). A *p*-value of <0.05 was considered statistically significant.

## Results

### Clinical Demographic and Clinical Data

A total of 9 patients who met the inclusion criteria were screened for detailed medical information (Table 1). The cohort comprised seven males and two females, with ages ranging from 15 to 45 years. The duration of the episode varied, with a minimum of 0.5 months and a maximum of 84 months. The daily paliperidone dosages included 3 mg (*N* = 1), 9 mg (*N* = 6), and 12 mg (*N* = 2). The mean blood drug concentration was 36.96 ± 6.72 ng/mL (*N* = 8). The data for age and episode duration were not normally distributed, while the blood drug concentration data followed a normal distribution.

**Table 1.** **Demographic and clinical data of the included patients**.

Patient number	Gender	Age	Episode duration	Maintaining daily dosage	Blood drug concentration	D-dimer levels before the treatment	D-dimer levels after the treatment
		(years)	(month)	(mg)	(ng/mL)	(µg/L FEU)	(µg/L FEU)
1	Male	34	48	9	34.6	170	170
2	Male	17	0.5	9	37.4	490	550
3	Male	21	12	3	*NA*	500	440
4	Male	15	1	9	26.1	230	290
5	Male	18	3	12	41.4	280	210
6	Male	16	4	9	38.0	170	170
7	Male	16	12	9	28.5	170	170
8	Female	45	84	12	49.3	250	260
9	Female	15	12	9	37.3	280	170

Abbreviation: NA, not available; FEU, fibrinogen equivalent unit.

### Levels of Serum D-dimer Before and After Paliperidone Treatment in Patients

**Fig. 1.**
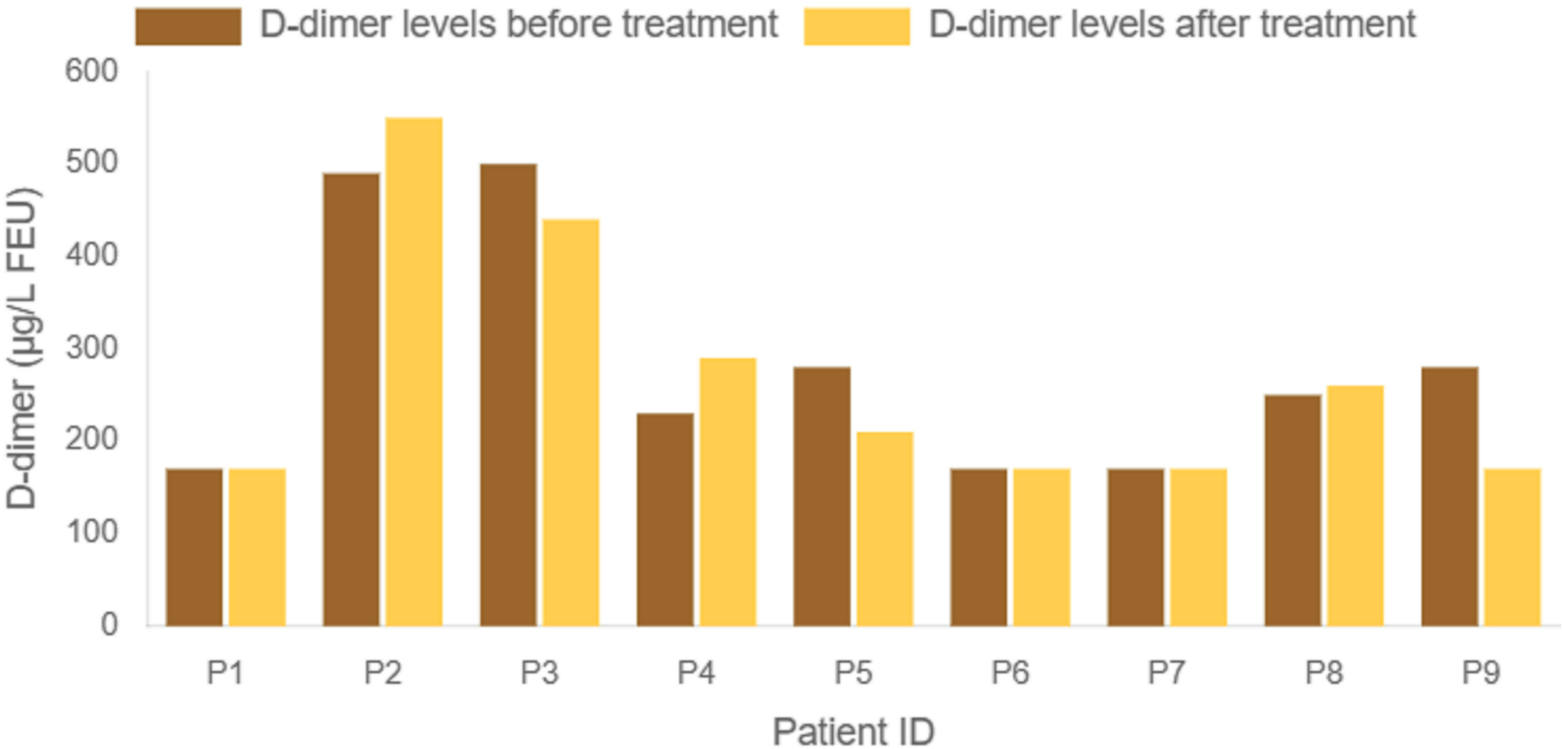
**Comparison of serum D-dimer levels before (brown) and after (orange) 2 weeks of paliperidone treatment**. FEU, fibrinogen equivalent unit.

All 9 patients had available serum D-dimer level data (Table 1), which showed a non-normal distribution according to the results of Shapiro-Wilk test (*p <* 0.05). Given the non-normal distribution of the data, the Wilcoxon Signed-Rank Test was applied. Overall, no significant difference was observed in D-dimer levels before and after two weeks of paliperidone treatment (n = 9, W = 12.5, *p* = 0.469) (Fig. 1).

### Effects of Paliperidone Treatment on Serum D-dimer Levels in Mice

**Fig. 2.**
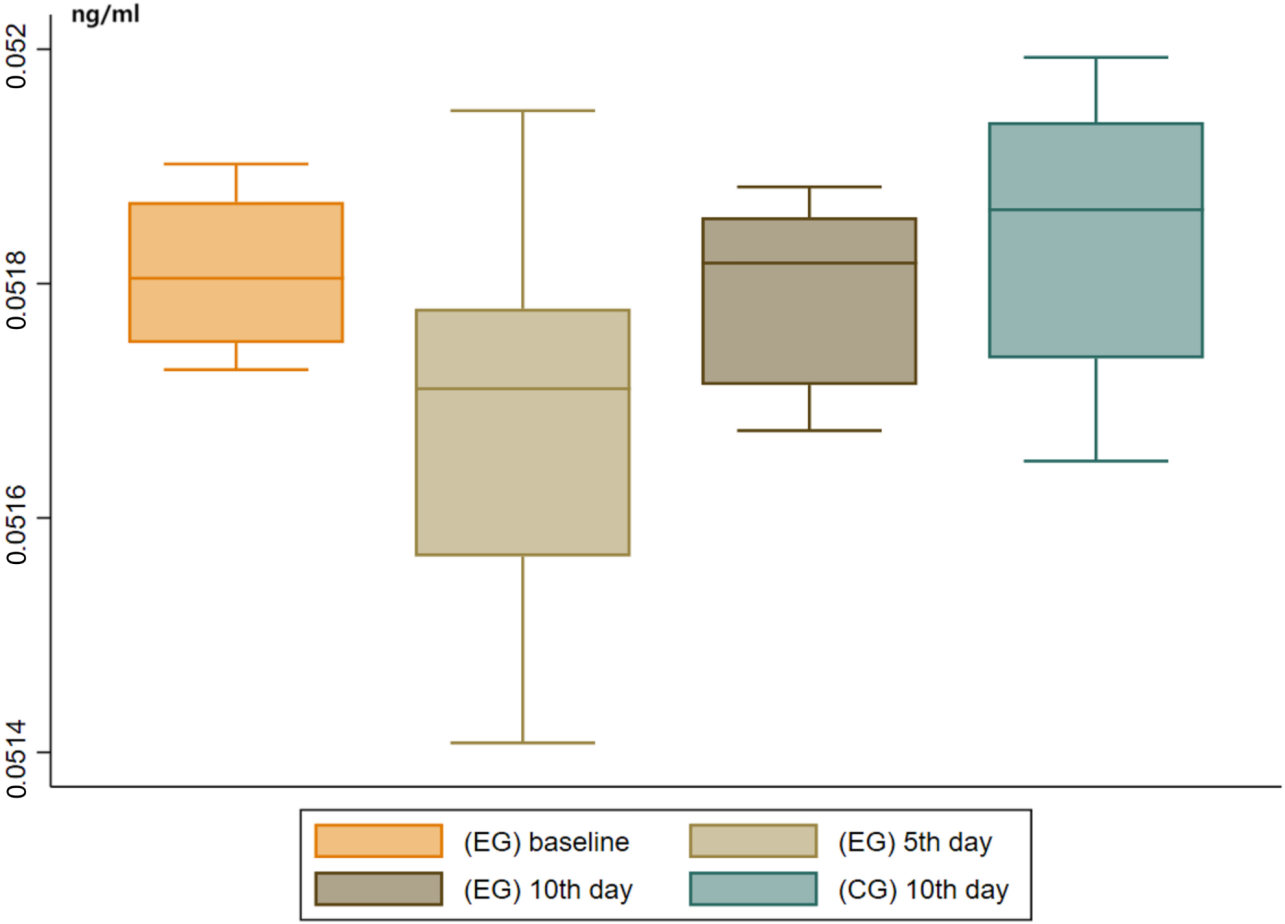
**Box plots of serum D-dimer levels for mice in the EG (baseline, 5th day, and 10th day after the start of paliperidone treatment) and the CG (10th day after the treatment began in the EG)**. The lines within the box represent the median, while the outer lines indicate the 95% confidence interval. EG, experimental group; CG, control group.

The Shapiro-Wilk test was used to assess normality. After removing an extreme value in EG on the 10th day, the remaining data followed a normal distribution (*p* > 0.05). In the EG, the serum D-levels were measured at baseline, the 5th day, and the 10th day. As for the CG we only measured the 10th day levels for reference. The independent sample *t*-test was employed to compare serum D-dimer levels between each EG point and the CG on the 10th day. Compared to the CG on the 10th day, serum D-dimer levels in the EG on the 10th day showed no significant difference (*p* > 0.05), while the level in the EG on the 5th day was significantly lower (*p* < 0.05). One-way repeated measures ANOVA and post-hoc tests were performed to compare intra-group differences within the EG. Compared to its baseline, serum D-dimer levels within the EG on the 5th day was significantly decreased (*p* < 0.05) (Table 2, Fig. 2).

**Table 2.** **Raw data of animal experiments**.

Group	(EG) Baseline		(EG) 5th day		(EG) 10th day		(CG) 10th day	
Item	OD value	Concentration (ng/mL)	OD value	Concentration (ng/mL)	OD value	Concentration (ng/mL)	OD value	Concentration (ng/mL)
Sample No.1	0.281	0.0517265	0.232	0.0514080	0.145	0.0508425^a^	0.269	0.0516485
Sample No.2	0.290	0.0517850	0.288	0.0517720	0.273	0.0516745	0.282	0.0517330
Sample No.3	0.283	0.0517395	0.269	0.0516485	0.301	0.0518565	0.309	0.0519085
Sample No.4	0.308	0.0519020	0.277	0.0517005	0.280	0.0517200	0.283	0.0517395
Sample No.4	0.306	0.0518890	0.315	0.0519475	0.295	0.0518175	0.310	0.0519150
Sample No.5	0.286	0.0517590	0.280	0.0517200	0.279	0.0517135	0.295	0.0518175
Sample No.6	0.300	0.0518500	0.244	0.0514860	0.297	0.0518305	0.322	0.0519930
Sample No.7	0.296	0.0518240	0.290	0.0517850	0.305	0.0518825	0.317	0.0519605
Mean ± SD	/	0.051809 ± 0.000067	/	0.051683 ± 0.000171*^/#^	/	0.051785 ± 0.000081	/	0.051839 ± 0.000124

^a^This extreme value was removed prior to data analysis. *p < 0.05 when compared to the baseline levels within the EG group; ^#^*p* < 0.05 when compared to the 10th day levels in the CG group. Abbreviations: EG, experimental group; CG, control group; OD, optical density; SD, standard deviation.

## Discussion

This study examined serum D-dimer levels in patients with schizophrenic spectrum disorders and wild-type C57 male mice. In these patients, D-dimer levels showed minimal changes after two weeks of paliperidone treatment. Similarly, in mice, a 10-day regimen of 3 mg/kg paliperidone daily did not lead to significant changes in serum D-dimer levels. However, after 5 days of treatment, serum D-dimer levels in EG showed a slight decrease compared to baseline and to CG on the 10th day.

Although several case reports have highlighted the potential risk of thromboembolic events induced by antipsychotics [11, 15, 16, 17], the current evidence remains inconclusive due to the lack of large cohort studies. Additionally, the observed effects of paliperidone on thrombosis appear to be limited to cellular changes, which are unlikely to result in significant clinical symptoms. For example, one study showed that paliperidone reduced epinephrine- and serotonin-induced human platelet aggregation in human plasma, but minimal changes were observed in platelet function, plasma coagulation, or fibrinolysis [18]. Therefore, our findings align with the current body of evidence.

However, the effects of paliperidone (and its precursor, risperidone) on coagulation function have raised growing concerns, underscoring the need for heightened awareness in the pharmacotherapy of schizophrenic spectrum disorders. Antipsychotic exposure is increasingly recognized as a potential risk factor for venous thromboembolism [9]. Previous studies suggest that paliperidone’s antagonistic effects on dopamine D2 receptors, prolactin elevation, and subsequent platelet aggregation and adhesion may play a role [19]. However, these hypotheses remain speculative due to several challenges: (1) changes in prolactin levels and D-dimer elevations are not observed in all cases; (2) patients with schizophrenic spectrum disorders who are receiving paliperidone may have other underlying factors contributing to thromboembolic risks. In our animal experiments, serum D-dimer levels in the EG showed a slight decrease after five days of treatment, which contrasts with previous findings. However, after another five days of paliperidone treatment, serum D-dimer levels returned to baseline, and no significant difference was observed between the EG and CG. This suggests that paliperidone may temporarily influence coagulation function in individuals with no prior exposure to the drug, but this effect is likely susceptible to various internal and external factors.

Another notable aspect is the dosage. The potential link between different daily dosages of paliperidone and varying thrombotic outcomes has been debated for years. A previous case involving a 17-year-old adolescent with schizophrenia, who experienced a sudden elevation in serum D-dimer levels shortly after paliperidone therapy, suggested that the dosage may not be a determining factor, as reducing the daily dosage did not result in a corresponding decrease in D-dimer levels [11]. Similarly, a study examining the changes in serum coagulation factors VIII and IX in rats suggested that paliperidone palmitate could induce coagulation dysfunction in a dosage-independent manner [20]. Although our study did not specifically address different dosage gradients, the daily dosages for patients were tailored to their medical needs. In the animal experiments, all mice in EG received the same batch of paliperidone, with dosages adjusted based on body weight. This suggests that our study’s findings may not support the dosage-independent conclusion.

This study has several limitations. First, the sample sizes for both patients and animals were relatively small, restricting the ability to explore covariates such as sex, total disease duration, baseline medications, and daily dosages of paliperidone. Additionally, due to constraints on medical data access during the inpatient period and follow-ups, only serum D-dimer levels before and after the first two weeks of treatment were analyzed, limiting the ability to track dynamic changes. In CG, D-dimer levels were measured only on the 10th day, not at baseline or on the 5th day, which is another limitation. Since the mice in the CG received only water, their D-dimer levels remained relatively stable during the 10-day study. The study’s findings would have been more robust if baseline and 5th day levels had also been measured in the CG. Furthermore, the use of wild-type C57 mice rather than a schizophrenia model limits the relevance of the findings to clinical scenarios involving schizophrenia. This study primarily focused on the effects of paliperidone on D-dimer levels rather than its antipsychotic role. Further investigation is needed to explore whether animal models of schizophrenia exhibit inherent coagulation dysfunction. Future research should include larger cohorts, preclinical and clinical studies, detailed subgroup analyses, and the inclusion of schizophrenia model animals to better understand the relationship between antipsychotics and coagulation dysfunction, gain deeper insights into biological mechanisms, and refine strategies for the secondary prevention of thromboembolic diseases.

## Conclusion

In conclusion, short-term paliperidone treatment did not result in significant changes in serum D-dimer levels. However, heightened attention to the potential risk of thrombosis in patients receiving antipsychotics remains advisable. Future research should aim to expand sample sizes and observation periods, with the inclusion of subgroup analyses for deeper insights.

## Availability of Data and Materials

All data generated or analyzed during this study are included in the manuscript.

## References

[1] Ebisch SJH, Salone A, Ferri F, De Berardis D, Romani GL, Ferro FM (2013). Out of touch with reality? Social perception in first-episode schizophrenia. *Social Cognitive and Affective Neuroscience*.

[2] McCutcheon RA, Reis Marques T, Howes OD (2020). Schizophrenia-An Overview. *JAMA Psychiatry*.

[3] Divac N, Prostran M, Jakovcevski I, Cerovac N (2014). Second-generation antipsychotics and extrapyramidal adverse effects. *BioMed Research International*.

[4] De Hert M, Yu W, Detraux J, Sweers K, van Winkel R, Correll CU (2012). Body weight and metabolic adverse effects of asenapine, iloperidone, lurasidone and paliperidone in the treatment of schizophrenia and bipolar disorder: a systematic review and exploratory meta-analysis. *CNS Drugs*.

[5] Şengül MCB, Kaya K, Yilmaz A, Şengül C, Serinken M (2014). Pulmonary thromboembolism due to paliperidone: report of 2 cases. *The American Journal of Emergency Medicine*.

[6] Michaud I, Landry P (2018). Case Report: Paliperidone Palmitate, But Not Aripiprazole, as a Possible Risk Factor for Pulmonary Embolism. *Journal of Clinical Psychopharmacology*.

[7] Yan Y, Wang L, Yuan Y, Xu J, Chen Y, Wu B (2024). A pharmacovigilance study of the association between antipsychotic drugs and venous thromboembolism based on Food and Drug Administration Adverse Event Reporting System data. *Expert Opinion on Drug Safety*.

[8] Huang J, Zou F, Zhu J, Wu Z, Lin C, Wei P (2024). Association between antipsychotics and pulmonary embolism: a pharmacovigilance analysis. *Expert Opinion on Drug Safety*.

[9] Ogłodek EA, Just MJ, Grzesińska AD, Araszkiewicz A, Szromek AR (2018). The impact of antipsychotics as a risk factor for thromboembolism. *Pharmacological Reports: PR*.

[10] Zheng C, Liu H, Tu W, Lin L, Xu H (2023). Hypercoagulable state in patients with schizophrenia: different effects of acute and chronic antipsychotic medications. *Therapeutic Advances in Psychopharmacology*.

[11] Wu C, Lai J, Ma S, Huang M, Hu S, Xu Y (2020). Paliperidone-Related Sudden Elevation of Plasma D-Dimer Levels: A Case Report. *Journal of Clinical Psychopharmacology*.

[12] Adam SS, Key NS, Greenberg CS (2009). D-dimer antigen: current concepts and future prospects. *Blood*.

[13] Schol-Gelok S, van der Hulle T, Biedermann JS, van Gelder T, Klok FA, van der Pol LM (2018). Clinical effects of antiplatelet drugs and statins on D-dimer levels. *European Journal of Clinical Investigation*.

[14] Tonne B, Pedersen MH, Fronas SG, Jorgensen CT, Amundsen EK, Maehlum JB (2023). Evaluation of the diagnostic performance of three D-dimer assays in patients with suspected deep vein thrombosis: STA-Liatest D-Di plus, Tina-quant D-dimer Gen. 2, and INNOVANCE D-dimer. *Thrombosis Update*.

[15] Konnakkaparambil Ramakrishnan K, George M (2021). Deep vein thrombosis on the fourth day of risperidone therapy. *BMJ Case Reports*.

[16] Sheikhmoonesi F, Bahari Saravi SF (2012). Deep venous thrombosis and atypical antipsychotics: three cases report. *Daru: Journal of Faculty of Pharmacy, Tehran University of Medical Sciences*.

[17] Jullian-Desayes I, Roselli A, Lamy C, Alberto-Gondouin MC, Janvier N, Venturi-Maestri G (2015). Rhabdomyolysis with Acute Renal Failure and Deep Vein Thrombosis Induced by Antipsychotic Drugs: A Case Report. *Pharmacopsychiatry*.

[18] De Clerck F, Somers Y, Mannaert E, Greenspan A, Eerdekens M (2004). In vitro effects of risperidone and 9-hydroxy-risperidone on human platelet function, plasma coagulation, and fibrinolysis. *Clinical Therapeutics*.

[19] Anaforoglu I, Ertorer ME, Kozanoglu I, Unal B, Haydardedeoglu FE, Bakiner O (2010). Macroprolactinemia, like hyperprolactinemia, may promote platelet activation. *Endocrine*.

[20] Yılmaz ED, Motor S, Sefil F, Pınar N, Kokacya H, Kisa M (2014). Effects of paliperidone palmitate on coagulation: an experimental study. *TheScientificWorldJournal*.

